# CD44-mediated activation of α5β1-integrin, cortactin and paxillin signaling underpins adhesion of basal-like breast cancer cells to endothelium and Fibronectin-enriched matrices

**DOI:** 10.18632/oncotarget.5461

**Published:** 2015-10-02

**Authors:** Suzanne McFarlane, Cheryl McFarlane, Nicola Montgomery, Ashleigh Hill, David J.J. Waugh

**Affiliations:** ^1^ Centre for Cancer Research and Cell Biology, Queen's University Belfast, Belfast, Northern Ireland, BT9 7BL

**Keywords:** CD44, breast cancer, Fibronectin, integrin, vascular endothelium

## Abstract

CD44 expression is elevated in basal-like breast cancer (BLBC) tissue, and correlates with increased efficiency of distant metastasis in patients and experimental models. We sought to characterize mechanisms underpinning CD44-promoted adhesion of BLBC cells to vascular endothelial monolayers and extracellular matrix (ECM) substrates. Stimulation with hyaluronan (HA), the native ligand for CD44, increased expression and activation of β1-integrin receptors, and increased α5-integrin subunit expression. Adhesion assays confirmed that CD44-signalling potentiated BLBC cell adhesion to endothelium and Fibronectin in an α5B1-integrin-dependent mechanism. Co-immunoprecipitation experiments confirmed HA-promoted association of CD44 with talin and the β1-integrin chain in BLBC cells. Knockdown of talin inhibited CD44 complexing with β1-integrin and repressed HA-induced, CD44-mediated activation of β1-integrin receptors. Immunoblotting confirmed that HA induced rapid phosphorylation of cortactin and paxillin, through a CD44-dependent and β1-integrin-dependent mechanism. Knockdown of CD44, cortactin or paxillin independently attenuated the adhesion of BL-BCa cells to endothelial monolayers and Fibronectin. Accordingly, we conclude that CD44 induced, integrin-mediated signaling not only underpins efficient adhesion of BLBC cells to BMECs to facilitate extravasation but initiates their adhesion to Fibronectin, enabling penetrant cancer cells to adhere more efficiently to underlying Fibronectin-enriched matrix present within the metastatic niche.

## INTRODUCTION

Extravasation, a critical step within the metastatic cascade concerns the arrest and adherence of circulating tumor cells (CTCs) on the vascular endothelium within distant tissues and organs. This process requires an initial arrest on the endothelial cells after which the adherent tumor cells traverse the endothelial barrier to gain access to the stroma of the underlying tissue. Tumor cells are proposed to replicate lymphocyte behavior and roll along the endothelial surface prior to attaching firmly to the endothelial monolayer [1]. As in lymphocyte adhesion, cancer cell extravasation is considered to be dependent upon multiple receptor dependent interactions between the tumor cell and the endothelium to regulate “rolling” and “arrest” on the endothelium, prior to exit of the cell from the circulation [2]. Moreover, in order for the invading cancer cell to form a clinically-relevant tumor within the new tissue, the cancer cell must embed and adhere to the substrates present within this new niche.

CD44 is a cell-surface glycoprotein receptor for hyaluronan (HA) and is an accepted marker for isolating tumourigenic, stem-like breast cancer cells [3, 4]. CD44 expression is selectively over-expressed in estrogen receptor (ER)- and progesterone receptor (PR)-negative breast tumors, with enrichment detected in basal-like breast cancer (BLBC) [5–7]. Furthermore, CD44-positive, stem-like breast cancer cells have been detected within the bone marrow of early-stage breast cancer patients [8, 9]. Our prior observations demonstrate the importance of CD44 in potentiating the adhesion of representative cell-based models of BLBC to human bone marrow endothelial cells (BMECs) *in vitro*, indicating a role in supporting more efficient extravasation of the cells [10, 11]. Moreover, we have shown that CD44 expression increases the efficiency of distant metastasis of BLBC cells *in vivo* [7]. Knockdown of CD44 reduced the incidence and size of distant metastases resulting from the intracardiac injection of BLBC cells, including reduced metastasis in the bone, lungs, liver and brain.

CD44 initiated adhesion has been shown to induce an integrin receptor-mediated adhesion of *T*-lymphocytes [12, 13], mediated in part through the complexing of CD44 with the α4β1-integrin receptor expressed on the surface of these cells, and secondly, through a downstream promotion of cytoskeletal reorganization [14]. In addition, Fujisaki and colleagues determined that CD44 signaling could initiate LFA-1 integrin activation to facilitate adhesion to endothelium and promote transactivation of colorectal cancer cells [15]. The objective of this study was to characterize the potential mechanism by which CD44 potentiates the adhesion of BLBC cells to endothelium and matrix substrates. Here we characterize the biochemical and functional importance of a CD44-induced regulation of β1-integrin receptor expression and activation in BLBC cells, resulting in the activation of a cortactin-paxillin signal transduction pathway which underpins cancer cell adhesion to endothelial cells and extracellular matrix (ECM).

## RESULTS

### CD44 signaling regulates specific α and β1-integrin expression

**Figure 1:**
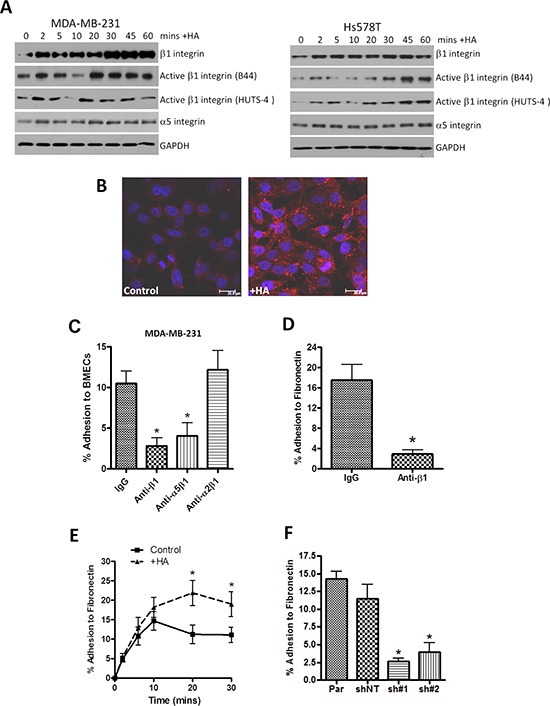
HA signaling promotes activation of β1-integrin receptors and promotes β1-integrin-mediated cell adhesion **A.** Western blots comparing time-dependent expression and changes in integrin activation status of β1-integrin subunits in MDA-MB-231 and Hs578T breast cancer cell lines following HA stimulation over a 60 min timecourse. Equal protein loading is confirmed by reanalysis of GAPDH expression. **B.** Representative fluorescence micrographs showing increased cell-surface immunoreactivity against the activated β1-integrin conformation (detected by B44 clonal antibody) following HA addition to MDA-MB-231BO cells for 20 min. Scale bar equivalent to 20 μm. **C.** Bar graph illustrating how administration of neutralizing antibodies to specific integrin chains or heterodimers modulates the efficiency of metastatic MDA-MB-231 cell adhesion over 30 min to a monolayer of human bone marrow endothelial cells (hBEMCs). fibronectin-coated plate. Antibodies were used at the concentration of 5 μg/ml, as recommended by the manufacturer. **D.** Bar graph illustrating how administration of a neutralizing antibody against β1-integrin receptors modulates the efficiency of metastatic MDA-MB-231 cell adhesion over 30 min to a Fibronectin-coated plate. **E.** Bar graph illustrating how stimulation of MDA-MB-231 cells with HA potentiates cell adhesion to Fibronectin over a 30 min timecourse. **F.** Bar graph illustrating the comparative adhesion of parental (Par) and CD44-depleted MDA-231 cells to a Fibronectin-coated plate over a 30 min period. Expression of CD44 levels in shNT (non-targeting control) and CD44-targeted (sh#1 and ch#2) cells following stable depletion are presented in McFarlane et al. 2015. All data shown in adhesion experiments are a mean ± SEM of at least 3 independent experiments. Statistically significant differences in adhesion were determined by a Student's *t*-test; *p < 0.05. Immunoblots are representative of three or more independent experiments.

α4β1-integrin receptors play an important role in the extravasation of CD44-positive *T*-lymphocytes *in vivo* [13]. We conducted experiments to characterize the relationship between CD44 and integrin subunit expression and/or activation, using two representative CD44-expressing models of BLBC, the MDA-MB-231 and Hs578T cell lines [6], and the metastatic prostate cancer cell line, PC3 [10]. Stimulation with low molecular weight HA (LMW-HA, the signaling ligand for CD44) promoted a rapid increase in β1-integrin subunit expression, together with an increased pool of activated β1-integrin receptors as detected by the B44 and HUTS-4 antibodies (that only recognize the active conformation of the β1-integrin) [16] (Figure 1A). Furthermore, immunofluorescence-microscopy confirmed the increased activated β1-integrin receptor pool post-HA stimulation in the MDA-MB-231 cells (Figure 1B). Although the α4-integrin subunit is proposed to mediate CD44-promoted adhesion of *T*-lymphocytes, we failed to detect expression of the α4-integrin subunit in BLBC cells by immunocytochemistry, immunoblotting or flow cytometry (data not shown). Instead, immunoblotting confirmed that HA stimulation of BLBC cells promoted a rapid increase in the expression of the α5-integrin subunit, concurrent with increased expression of the β1-integrin receptor chain (Figure 1A). A similar profile of increased expression and activation of the α5 and β1-integrin receptors was also detected in CD44-positive PC3 cells, a bone-metastatic prostate cancer line, in response to HA (Supplementary Figure S1A).

### Integrin receptors contribute to CD44 promoted cell-cell and cell-matrix adhesion

The importance of β1-integrin receptors in underpinning CD44-promoted adhesion to BMEC monolayers was studied using pan- or selective function-blocking integrin antibodies. Blockade of all potential β1-integrin heterodimers and specific inhibition of the α5β1-integrin receptor attenuated MDA-MB-231 cell adhesion to BMECs by 73% (*p* < 0.05) and 61% (*p* < 0.01), respectively. In contrast, α2β1-integrin blockade had no effect on MDA-MB-231 cell adhesion to BMECs (Figure 1C). A similar importance of the α5β1-integrin receptor was observed in PC3 cells (Supplementary Figure S1B).

### CD44 signaling promotes adhesion to fibronectin

The native ECM ligand of the α5β1-integrin heterodimer is Fibronectin. Therefore, we determined whether CD44-induced activation of this integrin may also underpin increased adhesion of MDA-MB-231 cells to this ECM substrate. Initial experiments demonstrated that pre-treatment with the β1-integrin function-blocking antibody reduced MDA-MB-231 adhesion to Fibronectin by 84% (*p* < 0.05), confirming the importance of β1-integrin receptors in mediating adhesion of CD44-positive MDA-MB-231 cells to Fibronectin (Figure 1D). The importance of CD44 signaling in promoting adhesion to Fibronectin was demonstrated in two further assays. Firstly, the addition of HA markedly increased the maximal adhesion of CD44-positive MDA-MB-231 cells to Fibronectin (*p* < 0.05) (Figure 1E). Furthermore, using stable CD44-depleted clones of MDA-MB-231 cells, we confirmed that loss of CD44 correlated with a significant decrease in adhesion potential to Fibronectin, reducing adhesion to approximately 20% of control values (*p* < 0.05) (Figure 1F).

### Bone-tropic breast cancer cells have increased pools of activated integrin receptors and demonstrate increased adhesion properties

**Figure 2:**
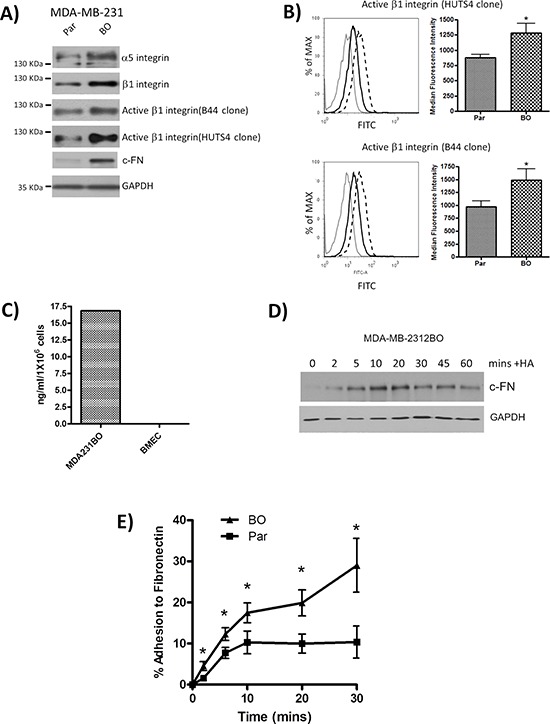
Characterization of bone-tropic metastatic breast cancer cells and their adhesion to Fibronectin **A.** Immunoblots comparing the expression and activation of integrin receptor chains and cellular fibronectin (c-FN) in parental (Par) and bone tropic (BO) clones of the MDA-MB-231 cells. Equal protein loading was confirmed by assessment of GAPDH expression. Immunoblots are representative of three or more independent experiments. **B.** Flow cytometry profiles illustrating the elevated expression of activated β1-integrin receptor pool using both B44 and HUTS-4 antibodies in MDA-MB-231BO cells relative to parental cells. Grey lines represent the isotype control, solid black lines represent profiles on parental MDA-MD-231 cells and the dashed lines represent profiles determined on MDA-MD-231BO cells. Inset bar graphs provide quantitative analysis of four independent profiling analyses. **C.** Bar graph presenting a representative profile of elevated FN secretion measured by specific analysis of conditioned media harvested from cultures of bone-tropic MDA-MB-231BO cells and BMECs. Data shown is representative of two independent experiments. **D.** Immunoblots comparing time-dependent changes in c-FN expression in MDA-MB-231 breast cancer cell lines following HA stimulation over a 60 min timecourse. Equal protein loading is confirmed by reanalysis of GAPDH expression. Immunoblots are representative of three or more independent experiments. **E.** Bar graph illustrating the comparative adhesion of parental (Par) and CD44-enriched bone tropic MDA-MB-231BO cells to a Fibronectin-coated plate over a 30 min period. Data shown in adhesion experiments are a mean ± SEM of at least 3 independent experiments. Statistically significant differences in adhesion were determined by a Student's *t*-test; *p < 0.05.

CD44 enhances the efficiency of distant metastasis *in vivo*, enabling the formation of tumors in multiple secondary sites including the lungs and skeleton. We have also observed that bone-tropic MDA-MB-231 (MDA-MB-231BO) cells expressed elevated levels of CD44 and adhered more rapidly to a BMEC monolayer *in vitro* [7]. Immunoblotting also reveals these CD44-enriched MDA-MB-231BO cells to express increased levels of the α5 and β1-integrin subunit relative to parental cells, and a greater pool of activated β1-integrin receptors (assessed using HUTS-4 and B44 antibodies) (Figure 2A). This was further confirmed by quantitative flow cytometry which detected an increased fluorescence intensity to the HUTS-4 and B44 antibodies in bone tropic cells (average of 33% more β1-integrins in the active conformation than parental cells) (*p < 0.05) (Figure 2B).

Further analysis was conducted to characterize Fibronectin expression between parental and bone homing clones of the MDA-MB-231 cell line. Consistent with increased CD44 expression and increased activation of the β1-integrin receptor pool, MDA-MB-231BO cells were shown to have elevated levels of cellular-Fibronectin (c-FN) (Figure 2A). Moreover, ELISA analysis confirmed a high level of Fibronectin secretion from MDA-MB-231 cells than BMECs (Figure 2C). MDA-MB-231BO cells were also stimulated with HA to determine any association of HA with FN expression in these cells. Immunoblotting confirmed that HA increased the expression of c-FN within these metastatic BLBC cells (Figure 2D).

Consistent with the increased production of Fibronectin and increased integrin activation detected on bone-tropic MDA-MB-231BO cells, these cells were also significantly more adherent to BMECs (reported in [7]) and Fibronectin than parental cells at all time points (*p* < 0.05) (Figure 2E).

### Talin facilitates CD44-induced β1-integrin activation in metastatic BLBC cells

**Figure 3:**
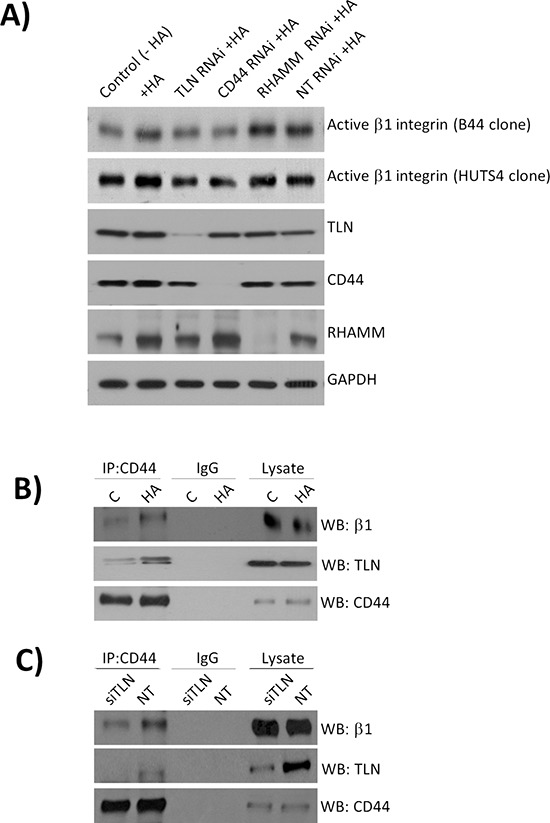
Characterization of mechanisms underpinning CD44-promoted integrin activation **A.** A panel of immunoblots characterizing siRNA-mediated knockdown of Talin (TLN), CD44 or RHAMM expression, and their subsequent effect on HA-induced activation of the β1-integrin receptor pool in MDA-MB-231 cells. Cells were stimulated with HA for 2 mins to focus understanding of the early events occurring downstream of CD44 activation. **B.** Immunoblots presenting the analysis of protein lysates harvested by co-immunoprecipitation experiments using anti-CD44 or anti-IgG antibodies (control). Cells were again stimulated with HA for 2 min to characterize the molecular interactions induced immediately downstream of CD44 initiated inside-out signalling. **C.** Representative blots of co-immunoprecipitation experiments characterizing the complex of CD44 with β1-integrins under HA stimulated (20 min) and talin-depleted conditions. Blots shown are representative of three independent experiments. The experimental design focused on understanding the importance of talin depletion on downstream integrin activation, thus introducing a longer incuation time. Immunoblots shown throughout the Figure are representative of three or more independent experiments.

Further experiments were conducted to characterize the mechanism underpinning CD44-promoted β1-integrin activation. Talin is a known interacting protein with the cytoplasmic tail of the β1-integrin. Using a series of gene-targeted siRNA strategies, we observed that knock-down of Talin attenuated the level of HA-induced β1-integrin activation in MDA-MB-231 cells, and was observed to be comparable to that observed in cells depleted of CD44 expression. Although HA stimulation increased expression of RHAMM, knockdown of this HA receptor using siRNA had no effect upon HA-stimulated activation of β1-integrin receptors (Figure 3A). In a further series of co-immunoprecipitation experiments, conducted in the absence and presence of a HA stimulus, we confirmed that both β1-integrin and Talin associate with CD44, and that the amount of these proteins associated with CD44 was potentiated following treatment with HA (Figure 3B). This protein complex was further studied under talin-depleted conditions. Transfection with talin siRNA reduced the level of β1-integrin in the anti-CD44 immunoprecipitate (Figure 3C). These results suggest that talin is a key intermediate protein facilitating CD44 promoted association with β1-integrin receptors and their activation.

### Cortactin and paxillin are key downstream signaling effectors of CD44/integrin signaling in BLBC cells

**Figure 4:**
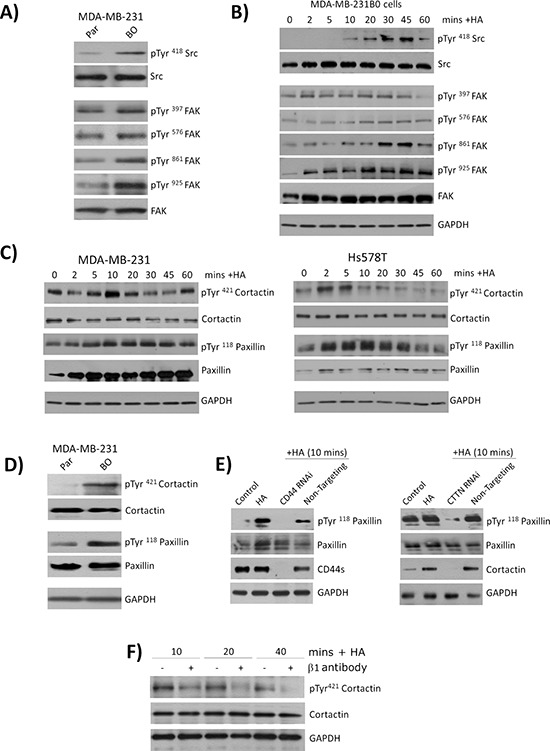
CD44 signaling promotes the phosphorylation of cytoskeletal proteins cortactin and paxillin **A.** Panel of immunoblots comparing the expression and phosphorylation status of the kinases Src and FAK in the parental and bone-tropic MDA-MB-231 cell lines. **B.** Immunoblots comparing the expression and phosphorylation status of Src and FAK following stimulation of MDA-MB-231BO cells with HA. **C.** Panel of immunoblots comparing the expression and phosphorylation status of the cytoskeletal proteins cortactin and paxillin in MDA-MB-231 (left panel) and Hs578T (right panel) breast cancer cell lines. **D.** Immunoblot comparing the basal expression and phosphorylation status of cortactin and paxillin in parental MDA-MB-231 and bone-tropic MDA-MB-231BO cells. **E.** Panel of immunoblots illustrating the levels of phosphorylation detected in paxillin in response to HA stimulation of MDA-MB-231 cells in the absence or presence of CD44 (left panel) or the absence and presence of cortactin (CTTN). **F.** Immunoblots measuring the levels of cortactin phospgorylation in HA-stimulated MDA-MB-2331 cells in the absence or presence of a neutralizing antibody to β1-integrin receptors. Equal protein loading in all immunoblots is confirmed by reanalysis of GAPDH expression. Blots shown in A-F are representative of at least three independent experiments.

Immunoblotting was performed to characterize HA-induced signaling pathways activated in BLBC cells. The non-receptor tyrosine kinases Src and FAK are both known substrates of integrin signaling and have confirmed roles in modulating cell adhesion, spreading and motility. Initial analysis confirmed increased phosphorylation of Src and FAK in MDA-MB231BO cells relative to parental cells (Figure 4A). Stimulation of MDA-MB-231 with HA activated both these kinases; a rapid onset and sustained phosphorylation of FAK was detected at multiple sites including the auto-phosphorylation site (Tyr397) and the integrin-receptor engagement site (Tyr861), the later reaching a maximal level of phosphorylation between 30 and 45 min post-stimulation (Figure 4B). Increased phosphorylation of Src was clearly apparent 10 min after HA stimulation.

We next examined the impact of HA stimulation upon the downstream substrates of FAK and Src, the cytoskeletal-associated proteins cortactin and paxillin. Immunoblotting analysis of MDA-MB-231, Hs578T (Figure 4C) and PC3 cells (Supplementary Figure S1C) confirmed a time-dependent induction of Tyr421 phosphorylation in cortactin following addition of exogenous HA. Similarly, HA addition increased the phosphorylation of paxillin in each of these BLBC cells. Interestingly, we also observed that HA reproducibly increased the expression of this cytoskeletal protein in both BLBC cells. Immunoblotting confirmed a higher level of constitutive phosphorylation of cortactin (Tyr421) and paxillin (Tyr118) in the bone-selective metastatic cells relative to the parental MDA-MB-231 counterpart (Figure 4D). The mechanism underpinning HA-induced phosphorylation of paxillin was studied further. MDA-MB-231BO cells were transfected with either a CD44-targeted or cortactin siRNA oligonucleotide pool. Depletion of CD44 or cortactin each abrogated the HA-induced phosphorylation of paxillin, detected in response to a 10 min exposure to HA (Figure 4E, left and right panels).

The importance of integrin activation to HA-induced phosphorylation of cortactin and paxillin was also studied, comparing HA-induced cytoskeletal protein phosphorylation in the absence and presence of a function-blocking β1-integrin antibody. Inhibition of β1-integrin signaling attenuated HA-induced phosphorylation of cortactin, detected 10, 20 and 40 min post-addition of the stimulus (Figure 4F).

### Cortactin and paxillin underpin CD44-promoted adhesion to endothelial monolayers and fibronectin

**Figure 5:**
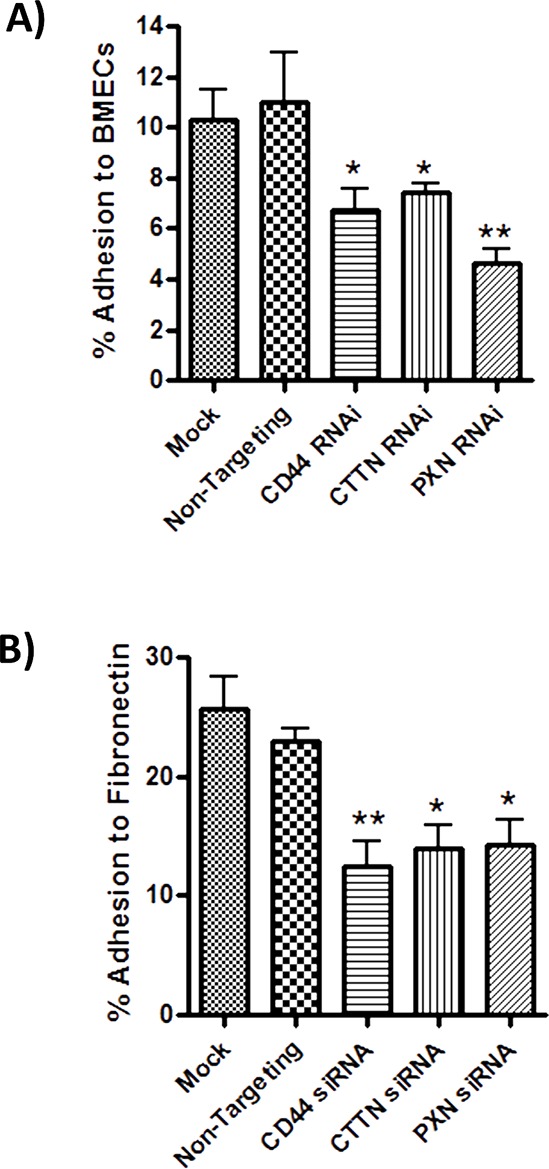
CD44 promoted cytoskeletal signaling mediates adhesion to endothelial monolayers and Fibronectin-enriched matrices **A.** Bar graph illustrating the effect of siRNA-mediated repression of CD44, cortactin (CTTN) or paxillin upon the adhesion of MDA-MB-231BO cells to BMECs. **B.** Bar graph illustrating the effect of siRNA-mediated repression of CD44, cortactin (CTTN) or paxillin (PXN) upon the adhesion of MDA-MB-231 cells to Fibronectin. The final concentration of siRNA-oligonucleotides used in all transfection reactions was 10 nM. Adhesion was measured 30 min post-addition of the cells to the substrate. All data is the mean ± S.E.M of three at least three independent experiments (*p < 0.05; calculated by two-tailed *t*-test analysis).

Adhesion assays were performed to characterize the relationship of HA-induced signaling events to the CD44- and integrin-promoted adhesion of tumor cells to BMECs and Fibronectin. Relative to the effect of a non-targeting siRNA, knock-down of CD44, cortactin and paxillin expression each reduced the magnitude of adhesion of MDA-MB-231 cells to BMECs by 39% (*p* < 0.05), 33% (*p* < 0.05) and 40% (*p* < 0.01), respectively (Figure 5A). Depletion of CD44, cortactin or paxillin expression also diminished adhesion of these cells to Fibronectin by 46%, 39% and 38% (*p < 0.05), respectively (Figure 5B). Treatment with an equivalent concentration of a non-targeting siRNA had no significant effect on adhesion of the cells to Fibronectin or BMECs.

## DISCUSSION

Our study provides further characterization of the importance of CD44 in augmenting the adhesion of malignant cells to the vascular endothelium. Our observations also extend our understanding of CD44-promoted adhesion, revealing a role for this receptor in initiating signaling events that promote cell adhesion to a matrix substrate Fibronectin, an ECM substrate reported to be enriched in the metastatic niche [17]. Using cell-based models of BLBC, we observe that exogenous HA administration initiates a cascade of CD44-dependent events that both promote the expression and activation of specific integrin receptors to mediate adhesion.

Biochemical and functional experiments demonstrate a complex inter-relationship of CD44 and the Fibronectin pathway. Firstly, immunoblotting and flow cytometry experiments confirm HA-induced CD44 signaling underpins a rapid increase in total and/or activated α5- and β1-integrin subunit expression in BLBC cells. The rapid changes in integrin expression that we observed may relate to characterized effect of CD44 signaling in activating downstream Akt, and Erk signaling [18], which can regulate the rate of protein translation [19, 20]. Secondly, we also confirm that HA can increase the synthesis of c-FN in these metastatic cells, at a time co-incident with the expression of the two integrin chains. This suggests that increased integrin receptor activation detected in HA-stimulated cells may in part relate to the promotion of “outside-in” signaling stemming from *de novo* Fibronectin synthesis. Thirdly, prior studies have reported that the cytoplasmic tail of CD44 is required for β1-integrin activation [21]. In co-immunoprecipitation experiments, we demonstrate that exogenous HA increases the association of talin with CD44, and the association of CD44 with the β1-integrin chain. In addition, the formation of the CD44-integrin-talin complex is enhanced upon exposure to HA. Moreover, the depletion of talin attenuated HA-induced integrin activation, confirming the role of this protein in mediating an “inside-out” HA-induced activation of integrin receptors. The association of CD44, talin and β1-integrin receptors in a potential “ternary” complex may explain the rapidity with which HA can induce receptor activation and downstream signal transduction in order to underpin cellular adhesion and/or motility. Furthermore, it is intriguing to note that Fibronectin is also reported to be a ligand for CD44 receptors [22], suggesting that HA-initiated CD44 signalling may also potentiate a direct FN-CD44 interaction, in addition to stimulating an inside-out activation of integrin receptors to enhance FN engagement with breast cancer cells. In summary, our data and prior published observations suggest that CD44 signaling can increase the expression, promote rapid translocation [23], and induce activation of integrin receptors [21]. This multi-modal regulation of integrin biology would be consistent with a strategy employed by a cell to rapidly modulate its adhesion to a favorable substrate or interface.

Our observation that CD44 increases α5-integrin and β1-integrin expression is consistent with the increased efficiency of HA-stimulated malignant cells to augment adhesion not only to endothelial cells, but also to Fibronectin. Fibronectin is a major constituent of the pre-metastatic niche, and is present in both bone and lung metastases [17]. Other recent studies associate Fibronectin expression with increased tumor aggressiveness and poor clinical outcome in invasive breast cancers [24]. The capacity of HA and CD44 to promote synthesis of and adhesion to Fibronectin would be consistent with a more efficient colonization of the metastatic niche by invading BLBC cells and the promotion of adhesion-dependent outgrowth of secondary tumors. This improved adaptation to the microenvironment of the metastatic niche would be consistent with our recent observations that CD44 increases the efficiency of metastasis formed following systemic administration of MBA-MB-231 cells [7].

Understanding the downstream signaling pathways driving CD44 and integrin promoted adhesion to endothelial cells and matrix proteins may identify opportunities to prevent and/or reduce the efficiency of metastasis outgrowth. Phosphorylation of cortactin and paxillin were shown to be important downstream effectors underpinning the CD44-initiated, integrin-promoted cell-cell and cell-matrix adhesion of BLBC cells. Consistent with our observation that CD44 promotes distant tumor formation within the skeleton *in vivo* [7], the over-expression and increased phosphorylation of cortactin have also been shown to potentiate the frequency of bone metastasis arising from the inoculation of MDA-MB-231 breast cancer cells into athymic nude mice [25]. Moreover, our observation that two substrates of Src signaling, i.e. cortactin and paxillin, underpin CD44-promoted adhesion and the positive correlation of CD44 to distant metastasis, is intriguing given the enrichment of Src activity detected in breast cancers with high propensity to spread to the bone [26, 27].

This report provides further molecular evidence to support experimental data linking CD44 to a more efficient metastasis of breast cancer *in vivo* [7, 28]. Studies from our research group alone provide evidence that not only does CD44 enable adhesion to distant endothelium and secondary tumor niches by promoting integrin-dependent signaling, CD44 also facilitates local invasion through up-regulation of uPA, metalloproteinase and cathepsin proteolytic activity in BLBC cells [6]. Furthermore, we and others have detected elevation of CD44 in the poor prognostic basal subtype of breast cancer or stem-like tumor cells that have disseminated to the bone marrow [5–8]. In contrast, other recent immunohistochemical studies suggest that CD44 expression correlates with a favorable patient outcome [29]. Therefore, given the known complexity of this glycoprotein with respect to its structural biology and its biological diversity, and the distinct clinical outcomes that have been reported, CD44 is unlikely to have clinical utility as a singular prognostic marker. However, future studies should address whether the analysis of CD44, increased FN and α5β1-integrin receptor expression and a cohort of other CD44-affiliated metastasis-associated genes (e.g. cortactin, MT1-MMP, cathepsin K, and uPA), may provide a more robust prognostic signature to differentiate clinical outcome and predict patient outcome.

In summary, we demonstrate the capacity of HA-induced CD44-mediated signaling to increase the efficiency of integrin-mediated adhesion of metastatic breast cancer cells to vascular endothelium and Fibronectin-enriched matrices. The capacity to increase cell-cell and cell-matrix adhesion is consistent with our observations that CD44 increases the efficiency of distant metastasis of basal-like breast cancer *in vivo*. Further investigations are required to address the endothelial cell counter-ligands that engage the activated integrin-receptors detected on the surface of these FN-secreting, CD44-enriched BLBCs to facilitate endothelial cell arrest and adhesion.

## MATERIALS AND METHODS

### Antibodies and reagents

Primary antibodies for anti-CD44 (R&D systems, UK), anti-cortactin (Upstate Biotechnology, NY, USA), anti-paxillin, anti-phosphoTyr^118^-paxillin were obtained from Cell Signaling Technology (Beverly, MA), while anti-α5β1-integrin, anti-α2β1-integrin, anti-β1-integrin, activated-β1-integrin conformations (B44 and HUTS-4), cellular Fibronectin and phosphoTyr^421^-cortactin were purchased from Millipore (Watford, UK). HRP-conjugated secondary antibodies were obtained from Amersham, UK and Hyaluronan (MW220 kDa) from Lifecore Biomedical (MN, USA). Cells were stimulated with 100 μg/ml HA for indicated times. All other reagents were purchased from Sigma (Poole, UK) unless otherwise stated.

### Cell culture

Authenticated MDA-MB-231, MDA-MB-157 and PC3 lines were obtained from ATCC (Teddington, UK). Hs578T cells were kindly provided by Dr Paul Mullan (Centre for Cancer Research and Cell Biology, Queen's University Belfast). The highly invasive clone, MDA-MB-231BO was provided complete with matched parental cells by Prof. Toshiyuki Yoneda (University of Osaka, Japan) [30] and were cultured in DMEM supplemented with 10% v/v foetal calf serum (FCS) (Invitrogen™ Life Technologies, Paisley, UK). CD44 depleted luciferase-expressing MDA-MB-231 cells from Caliper Life Sciences (Cheshire, UK) were generated in our laboratory by stable transfection with CD44 shRNA or non-targeting shRNA control as previously described [7] and cultured in DMEM supplemented with 10% v/v FCS, 0.2 μg/ml puromycin. All cell lines were grown to 70% confluence prior to experimentation. Human bone marrow endothelial cells (BMEC) were provided by Dr Babette Weksler (Cornell University, NY) and cultured as previously described [10].

### Invasion and adhesion assays

Cell invasion and adhesion were assayed as described previously [6, 11].

### Flow cytometry

Cells were fixed in 1% paraformaldehyde and stained with HUTS4 and B44 antibodies according to manufacturer's instructions followed by detection with FITC-conjugated secondary antibody (Dako, Ely. UK). Data was acquired with a FACSCantoII (BD Biosciences, Oxford, UK) and analysed using FlowJo software. Isotype-controls were used to determine baseline parameters in these experiments.

### SDS-PAGE and western-blotting

Cell lysates (20 μg) were separated by SDS-PAGE and transferred onto immobilon-P PVDF membranes (Millipore, Billerica, MA, USA). Membranes were blocked for 1 h at RT in Tris-buffered saline/0.1% Tween-20 (TBS-T) containing 5%(v/v) dried milk. Incubation with primary antibody was carried out at 4°C overnight. Membranes were washed three times in TBS-T and then incubated for 1 h with the appropriate horse-radish peroxidase (HRP)-labeled secondary antibody. Following three washes in TBS-T, immunoreactivity was detected using SuperSignal West Pico Chemiluminescent Substrate (Pierce Biotechnology, Rockford, IL, USA) followed by exposure to X-ray film. Note, for detection of activated β1-integrins, lysates were electrophoresed on 7.5% SDS-PAGE gels under non-reducing conditions as detailed previously [16].

### Co-immunoprecipitation

1 mg cell lysate was incubated overnight at 4°C with 5 μg of mouse anti-CD44 or IgG2A (50 mM Tris [pH 8.0], 5 mM EDTA, 1% CHAPS, protease inhibitor cocktail). Immune complexes were precipitated using 20 μL sheep anti-mouse dynabeads (Invitrogen) for 2 h followed by three washes with immunoprecipitation buffer. Eluted samples and analyzed by Western-blotting for CD44, β1-integrin or Talin.

### Fluorescence microscopy

Cells fixed with 4% paraformaldehyde were stained according to antibody manufacturer's instructions and detected with TRITC–conjugated donkey anti-mouse (Jackson ImmunoResearch, Newmarket, UK). Nuclei were counterstained with 300 nmol/L DAPI (Molecular Probes, Life Technologies, Paisley, UK). Slides were mounted using Vectashield (Vector Laboratories, Peterborough, UK) and viewed with LSM 510 META NLO confocal microscope (Zeiss, Cambridge, UK). Isotype-controls were used to set baseline paremeters in these experiments.

### RNAi suppression of CD44, cortactin and paxillin

CD44/cortactin/paxillin expression was suppressed using validated RNAi oligonucleotide pools (SMARTpool^®^, GE Dharmacon, Lafayette, CO). Cells were transfected using DharmaFECT2 for 72 h prior to experimentation.

### Fibronectin ELISA

Fibronectin ELISA was obtained from Millipore and was used according to manufacturer's instructions.

### Statistical analysis

For all *in vitro* analysis, significance was analyzed using a two-tailed Student's *t*-test. Analysis was performed by comparing interventional arms to both untreated/unstimulated controls and the isotype-control arms, although statistical data reported in the manuscript relates to the isotype-control or vehicle control arms.

## SUPPLEMENTARY FIGURE


