# Supplementary material for: CD44-mediated activation of α5β1-integrin, cortactin and paxillin signaling underpins adhesion of basal-like breast cancer cells to endothelium and Fibronectin-enriched matrices
Source: Oncotarget. 2015 Oct 2;6(34):36762–73. doi: 10.18632/oncotarget.5461 (PMC4742209; doi:10.18632/oncotarget.5461)
Supplement: Supplementary file 1 [file oncotarget-06-36762-s001.pdf]

## SUPPLEMENTARY FIGURE

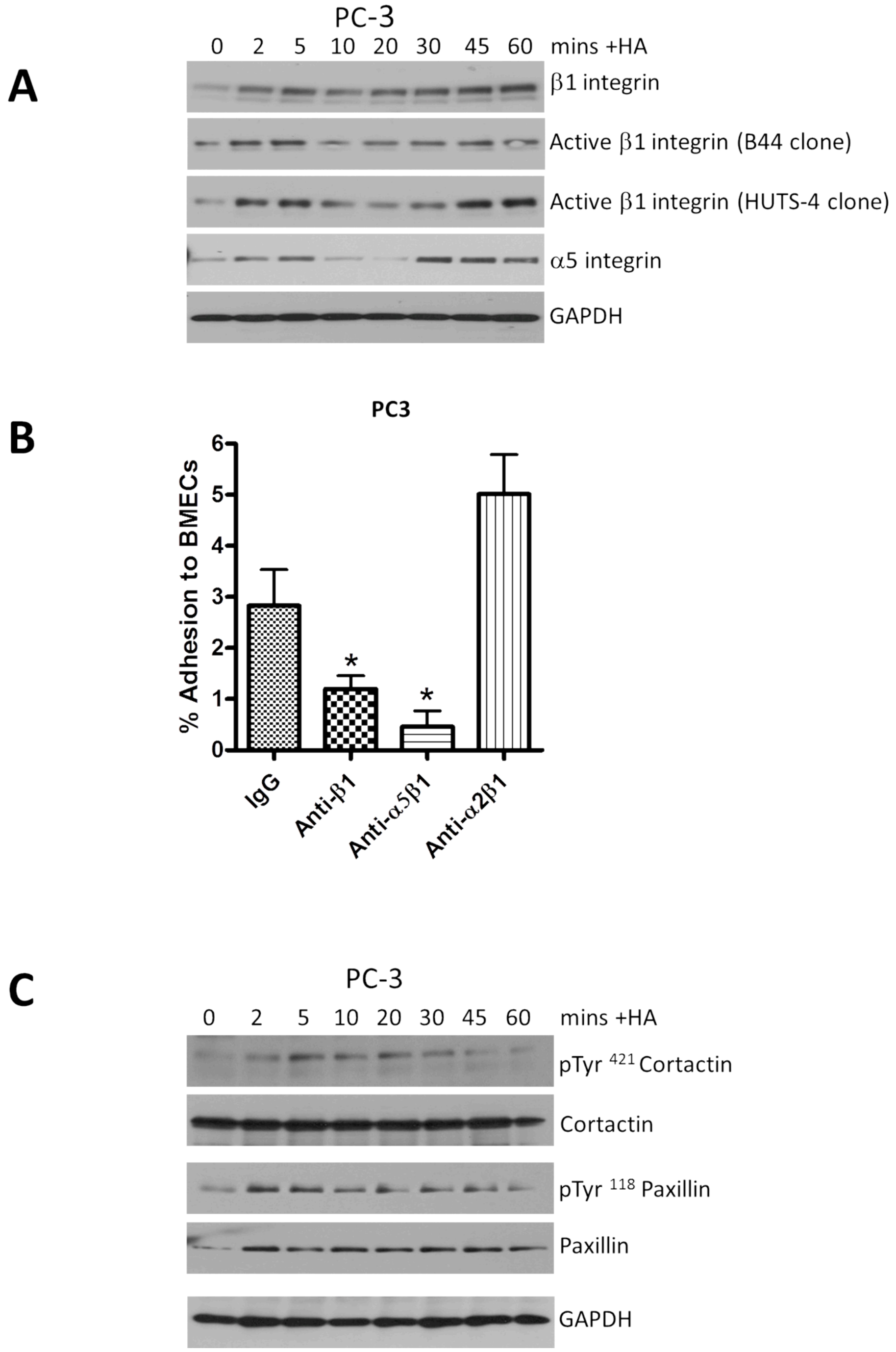


**Supplementary Figure S1: HA signaling promotes activation of β1-integrin receptors and promotes β1-integrin-mediated cell adhesion in prostate cancer cells. A.** Western blots comparing time-dependent expression and changes in integrin activation status of β1-integrin subunits in PC3 prostate cancer cell lines following HA stimulation over a 60 min timecourse. Equal protein loading is confirmed by reanalysis of GAPDH expression. **B.** Bar graph illustrating how administration of neutralizing antibodies to specific integrin chains or heterodimers modulates the efficiency of metastatic PC3 cell adhesion over 30 min to a monolayer of human bone marrow endothelial cells (hBEMCs). Antibodies were used at the concentration of 5 µg/ml, as recommended by the manufacturer. **C.** Panel of immunoblots illustrating the levels of phosphorylation detected in paxillin and cortactin in response to HA stimulation of PC3 cells. Equal protein loading in all immunoblots is confirmed by reanalysis of GAPDH expression. Blots shown are representative of at least three independent experiments.
